# Prevalence of Vitamin D Inadequacy in Urolithiasis Patients

**DOI:** 10.7759/cureus.15379

**Published:** 2021-06-01

**Authors:** Kunal Dholakia, Nivash Selvaraj, Narasimhan Ragavan

**Affiliations:** 1 Uro-oncologist, Apollo Hospitals, Chennai, IND; 2 Urology, Apollo Hospitals, Chennai, IND

**Keywords:** vitamin d, urolithiasis, parathyroid level, prevalence, inadequacy

## Abstract

Introduction

The role of vitamin D in kidney stone disease is unclear. Current evidence and existing studies are inconsistent and inconclusive. The objective of this study is to assess the prevalence of vitamin D (VD) inadequacy (VDI) and metabolic abnormalities in urolithiasis patients presenting to a tertiary care center.

Materials and methods

This is a prospective case-control study of 200 patients divided into two groups - Group 1: 100 urolithiasis patients (case group), and Group 2: 100 non-urolithiasis patients (control group) - which was conducted from January 2016 to January 2017. Demographic, clinical data, parathyroid level, serum 25-hydroxy VD [25(OH)D], and metabolic stone work-up were recorded and analyzed.

Results

Patient demographics were comparable in both groups. The prevalence of vitamin D inadequacy in urolithiasis patients was 95% as compared to 57% in the control group. The mean value ± SD of serum vitamin D in urolithiasis patients (16.5 ± 8.6 ng/mL) was significantly lower than in non-urolithiasis patients (28.7 ± 8.3 ng/mL) (p = <0.0001). Thirty-seven percent of the patients were recurrent stone formers. Hyperparathyroidism was observed in 77% of the patients and 71% of them were secondary to VDI.

Conclusion

Urolithiasis patients were found to have an increased prevalence of deficient VD related to secondary hyperparathyroidism.

## Introduction

Urolithiasis is a common, recurring, and multifactorial disease. The prevalence of urolithiasis in males is 10-15% and 3-5% in females [1]. In addition to its high prevalence, it also has a recurrence rate is of 50% at five years and 80-90% at 10 years [2]. Urolithiasis is a biochemical disease for which a surgical solution is being provided. Hence, it is important to investigate to understand the pathogenesis of urolithiasis, which will help possible prevention as well as avoidance of recurrent stone formation. Calcium-based stones are the commonest (>80%), and increased urinary excretion of calcium is a very strong predictor for stone formation. Urolithiasis patients tend to experience increased enteric calcium absorption, augmented urinary calcium excretion, and excessive bone mineral loss. Although direct actions of active vitamin D (VD) have been implicated in all these processes, the effect of VD (VD2 or VD3) on calcium homeostasis in urolithiasis is still not clear [3]. Very few studies have been performed to investigate the association of VD and urolithiasis, and their results are not clear [3-9]. It is not quite clear the precise levels of VD in urolithiasis patients, especially in the Indian context. We aimed to investigate the levels of VD in urolithiasis patients because it is an important component of calcium-phosphate homeostasis and may help us in understanding the pathogenesis of stone formation. This is a prospective case-control study to analyze the prevalence of VD inadequacy (VDI) in urolithiasis patients so as to assess whether it could be a risk for stone formation, and if it could help to establish a therapeutic intervention aimed at decreasing the risk of recurrence.

## Materials and methods

Study site and population

The study was conducted in a tertiary care hospital in a nonrandomized fashion using prospectively collected data. The target population enrolled were individuals who had documented proof of having urolithiasis assessed by radiological means - X-ray, ultrasound, or computed tomogram - irrespective of gender. The age-matched control population (non-urolithiasis patients, proved radiologically) were selected from individuals undergoing health check at the study site. Individuals <18 years of age, pregnant; with a known history of endocrine or metabolic causes of stone disease; with anatomical variations in the urinary tract (e.g., upper pelvic-ureteric junction obstruction, horseshoe kidney, and calyceal diverticula); on VD treatment or lithogenic drugs, and with a previous history of gastrointestinal surgeries or malabsorption syndromes were excluded from the study. The study was conducted over the period of one year and was reviewed and approved by the Apollo Institutional Ethics Committee - Clinical Studies (ECR/37/Inst/TN/2013).

Study format

All prospective patients with a documented proof of having urolithiasis (case group) and not having urolithiasis (control group) underwent extensive evaluation. Patient demographics, history of prior stones, medication history, and medical and surgical history were recorded. Subsequently, a biochemical assessment was performed, which included serum creatinine, serum calcium, serum uric acid, and serum VD3 levels, and their results were noted. Serum parathyroid hormone (PTH) was assessed only in urolithiasis patients. VDI was defined as level <30 ng/mL, of which insufficiency was defined as level 21-29 ng/mL and deficiency was defined as level <20 ng/mL. It was measured by Elecsys Roche® Electrochemiluminescence binding assay (Roche Diagnostics, Basel, Switzerland). The cases were compared with age- and gender-matched controls to validate the VD deficiency levels.

Sample size

The sample size was calculated using a formula that showed that 85 or more patients should be enrolled as cases, which were compared to a similar number of age-matched control population according to the allocation ratio of 1:1.

Sample size calculation

The following formula was used to calculate the sample size:

$N = \frac{Z^{2}pq}{d^{2}}$

where:

N = Sample size

Z = Standard normal variate value = 1.96 (95% confidence interval)

p = Prevalence of VD deficiency in stone patients (33.7% according to Elkoushy et al. [10])

q = 100-p

d = allowable error (5-20% of p) 

SPSS Statistics for Windows, version 22 (IBM Corp., Armonk, N.Y., USA) was used for the statistical calculation.

## Results

In our prospective observational comparative study, a total of 100 cases and 100 controls were studied.

Patient demographics

The mean age of the case group was 38.0 ± 11.8 years, which was not significantly different from the control group, whose mean age was 39.6 ± 11 years (p = 0.305). There was no gender bias between the groups (p = 0.102): the male-to-female ratio in the case group was 8:2, which was comparable to the control group's 7:3. Both the groups were comparable in terms of comorbid condition (such as systemic hypertension, type 2 diabetes mellitus, and chronic obstructive pulmonary disease) prevalent in them (p = 0.268). Eighty percent of the case population had an indoor occupation and the rest had an outdoor occupation. Thirty-five percent of the case group resided in Chennai (local), 54% resided outside Tamil Nadu but in India (national), and 11% lived outside India (international - from Middle Eastern countries). Thirty-seven percent of the patients had a history of urinary stone formation. The mean value (± SD) of serum VD level in the population working indoor was 16.55 ± 8.68 ng/mL, compared to the outdoor-working population's 16.31 ± 8.18 ng/mL (p = 0.966). The Indian population had statistically low levels of VD compared to the international patient population (p = 0.005).

Biochemical analysis

**Figure 1:**
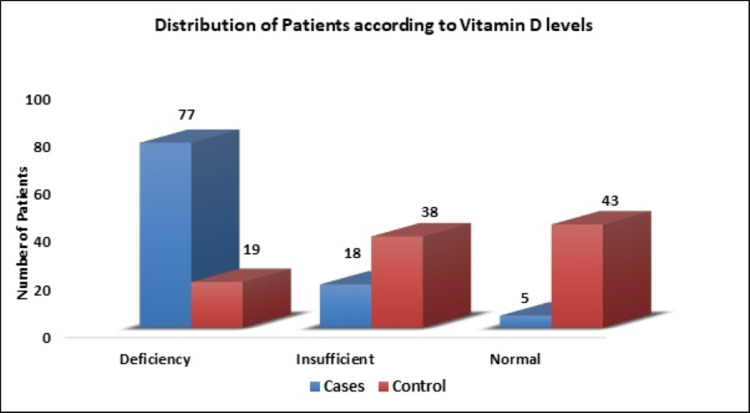
Distribution of patients according to vitamin D levels

The mean values of serum creatinine, serum calcium, and serum uric acid level in the case group were not statistically different from the control group. The prevalence of VDI in urolithiasis patients was 95% as compared to 57% in the control group. Seventy-seven percent of the patients had VD deficiency (≤20 ng/mL) and 18% patients had VD insufficiency (21-30 ng/mL) among urolithiasis patients. Among those who were not stone formers (SFs), 19% were deficient and 38% had insufficient serum VD levels. Figure 1 shows the distribution of patients according to VD levels.

The mean value ± SD of serum VD in urolithiasis patients (16.5 ± 8.6 ng/mL) was significantly lower than in the non-urolithiasis group (28.7 ± 8.3 ng/mL) (p ≤ 0.0001). Table *1* shows the characteristics of the two groups.

**Table 1:** Analysis of characteristics between two groups *Significant at p < 0.05

Parameters	Case (n=100)	Control (n=100)	P
Age (years), mean±SD	38.0±11.8	39.6±11	0.305
Gender (male:female)	8:2	7:3	0.102
Comorbidities (present in %)	9.0	5.0	0.268
Serum creatinine (mg/dl), mean±SD	0.9±0.26	0.93±0.26	0.353
Serum uric acid (mg/dl), mean±SD	5.7±1.5	5.6±1.4	0.799
Serum calcium (mg/dl), mean±SD	9.4±0.4	9.3±0.4	0.137
Serum Vitamin D (ng/dl), mean±SD	16.5±8.6	28.7±8.3	<0.0001*

The mean value ± SD of serum parathyroid level in urolithiasis patients was 91.86 ± 38.57 pg/mL. In patients with inadequate VD levels, 74.73% had increased serum parathyroid levels (normocalcemic secondary hyperparathyroidism due to VDI) and 25.26% had normal serum parathyroid levels. This difference was statistically significant (p ≤ 0.0001). Table 2 shows the comparison of serum parathyroid with respect to serum VD.

**Table 2:** Comparison of serum parathyroid with respect to serum vitamin D *Significant at p < 0.05; PTH=Parathyroid hormone

Parameters	Inadequate Vitamin D (n=95), n (%)	Normal Vitamin D (n=5), n (%)	P
Normal PTH	24 (25.26)	2 (40)	<0.0001*
Raised PTH	71 (74.73)	3 (60)	

## Discussion

There is a recent explosion in scientific literature that clearly documents the global prevalence of VD insufficiency as determined by low circulating levels of 25-hydroxy (25[OH]D) [11-13]. Factors contributing to the worldwide prevalence of VD insufficiency vary among countries, but in all cases, it involves limitations in either or both cutaneous synthesis and dietary sources of VD. The probable causes of VDI in the Indian population include darker skin having high melanin content (which acts as a natural sunscreen); social and/or religious norms related to public modesty (which dictate that most parts of an individual’s body should be covered); desk/sedentary jobs and confined, close spaces (which preclude adequate exposure to sunlight); low intake of dietary calcium; and traditional cooking practices (which degrade VD at high temperatures making it unavailable for consumption).

In our study, we assessed serum VD levels in 200 patients (SFs and non-SFs). Of the 200 patients, 152 (76%) had inadequate levels of VD. We also found that the Indian population (85%) had statistically low levels of VD compared to the international patient population (10%) (p = 0.005). This is similar to various studies that have highlighted 70-100% of Indians in different age groups as having VD insufficient or deficient levels [14].

Vitamin D and stones

In our study, of the 100 patients with urolithiasis, 95 (95%) patients had inadequate VD levels, out of which 77 (77%) were VD deficient and 18 (18%) were VD insufficient. The mean (± SD) VD level in urolithiasis patients was 16.5 ± 8.6 ng/mL, which was significantly lower than the levels in non-urolithiasis patients, which was 28.7 ± 8.3 ng/mL (p ≤ 0.0001). The evidence to prove the prevalence of VDI in urolithiasis patients was found in a similar study done by Elkoushy et al. [10], though the percentage of patients having VDI is much higher in our group. The other studies that showed similar findings were by Girón-Prieto et al., Pipili and Oreopoulos, and Roussos et al. [15-17].

There were a few analyses that did not corroborate our findings. Netelenbos et al. and Tang et al. in Colorado, USA showed that concentrations of serum 25(OH)D were not different between SFs and non-SFs [18,19]. There are also some studies that contradict our observations. Shakhssalim et al. and Hu et al. showed that SFs had significantly higher concentrations of 1,25(OH)2D than non-SFs [8,9]. These variations in results are because the studies have been conducted in different countries with vast variations in geographical, cultural, social, and economic conditions, as well as in the genetics of their populations. Our study is the first of its kind in the Indian population.

The significant relationship between urolithiasis and VD deficiency will allow researchers to postulate that there is a pathophysiological link between the two conditions. High levels of calcium reabsorption from the filtrate to blood or mild PTH increase could be involved [20]. VD deficiency would also cause phosphorus depletion, with a secondary increase in urinary calcium excretion and a decreased responsiveness to PTH [21]. It might also promote the onset of incomplete distal renal tubular acidosis (DRTA), a condition frequently associated with osteoporosis in adults and rickets in children [22]. Recurrent calcium nephrolithiasis will be associated with incomplete DRTA, although often unrecognized due to difficulty in diagnosis [23]. Interestingly, a study on animal models of nephrolithiasis shows that calcium and VD supplementation leads to a decrease of urinary calcium - instead of an increase - suggesting that VD deficiency could actually play a role in the formation of stones [24].

Parathyroid hormone levels and vitamin D deficiency

In the current study, we observed secondary hyperparathyroidism due to VDI, which was significantly higher as compared to patients with normal PTH levels in spite of inadequate VD levels (p ≤ 0.0001). The mean serum calcium level was within normal limits (9.4 ± 0.4 mg/dl). Since we had excluded the SFs with primary hyperparathyroidism, the disputed entity of normocalcemic hyperparathyroidism could be present. Our finding of normocalcemia with secondary hyperparathyroidism coincides with the results of other authors [25].

The reason behind the decreased levels of 25(OH)D and the association with PTH in SFs needs detailed study. The complexity of PTH and VD interactions has resulted in muscle and bone abnormalities in the general population [26]. This has been associated with the role of Klotho regulators of phosphate excretion, PTH, calcitriol synthesis, and fibroblast growth factor 23 (FGF-23) [27]. Decreased bone mass and high risk for osteoporotic fractures as a consequence of PTH and VD abnormalities have also been documented in calcium SFs. There is also a contributing role of FGF-23 and Klotho gene polymorphism in the pathogenesis of calcium nephrolithiasis [28-30]. Therefore, they are likely to influence the VD status in this patient population.

Our study has some inherent weaknesses. The study was not a randomized controlled one. Several unmeasured parameters potentially influencing VD homeostasis, such as serum phosphorus, were not measured. As this study is the first of its kind in India, a detailed metabolic evaluation (e.g., 24-hour urine analysis) was not feasible. Finally, stone analysis to know its composition could not be done.

## Conclusions

In this study, we have found that there is high prevalence of VDI with evidence of a significant secondary increase in serum parathyroid levels with normal serum calcium levels in urolithiasis patients. Further, a large-scale, multicenter study is recommended to reconfirm the association between VD and kidney stones. In addition, timely therapeutic intervention with VD could prevent urolithiasis in the population.
